# The SUMOylated METTL8 Induces R-loop and Tumorigenesis via m3C

**DOI:** 10.1016/j.isci.2020.100968

**Published:** 2020-03-07

**Authors:** Li-Hong Zhang, Xue-Yun Zhang, Tao Hu, Xin-Yun Chen, Jing-Jia Li, Manfred Raida, Ning Sun, Yan Luo, Xiang Gao

**Affiliations:** 1 Department of Biochemistry and Cancer Institute of the Second Affiliated Hospital, Zhejiang University School of Medicine, Hangzhou 310009, China; 2 Key Laboratory of Cancer Prevention and Intervention of China National Ministry of Education, Hangzhou 310009, China; 3 Department of Physiology and Pathophysiology, School of Basic Medical Sciences, Shanghai Medical College, Fudan University, Shanghai 200032, China; 4 Department of Spine Surgery, Shanghai East Hospital, Tongji University, Shanghai 200092, China; 5 Life Sciences Institute, National University of Singapore, Singapore 117456, Singapore

**Keywords:** Biological Sciences, Molecular Biology, Molecular Structure, Molecular Mechanism of Gene Regulation, Cancer

## Abstract

R-loops, three-stranded DNA-DNA:RNA hybrid structures, are best known for their deleterious effects on genome stability. The regulatory factors of this fundamental genetic structure remain unclear. Here, we reveal an epigenetic factor that controls R-loop stability. METTL8, a member of the methyltransferase-like protein family that methylates 3-methylcytidine (m3C), is a key factor in the R-loop regulating methyltransferase complex. Biochemical studies show that METTL8 forms a large SUMOylated nuclear RNA-binding protein complex (∼0.8 mega daltons) that contains well-reported R-loop related factors. Genetic ablation of METTL8 results in an overall reduction of R-loops in cells. Interaction assays indicated METTL8 binds to RNAs and is responsible for R-loop stability on selected gene regions. Our results demonstrate that the SUMOylated METTL8 promotes tumorigenesis by affecting genetic organization primarily in, or in close proximity to, the nucleolus and impacts the formation of regulatory R-loops through its methyltransferase activity on m3C.

## Highlights

• DNA:RNA hybrid structures are regulated by RNA methyltransferase via 3-methylcytidine
• SUMOylation stabilizes the RNA methyltransferase complex in the nucleus
• Dysregulation of DNA:RNA hybrids may induce tumorigenesis in mammalian cells

## Graphical Abstract

**Figure:**
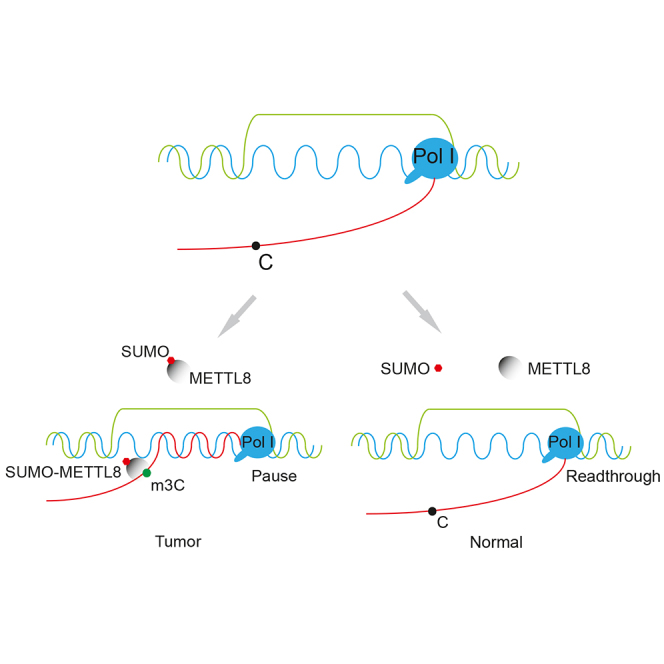

## Introduction

Epigenetic regulation is generally recognized to be related to covalent modifications of histones within chromatin or of genetic material such as DNA itself (Allfrey et al., 1964, Gold et al., 1963a, Gold et al., 1963b), which has powerful regulatory roles that impact the transcriptional landscape. Methyltransferase-like proteins (METTLs) share a well-conserved seven-beta strand s-adenosylmethionine (SAM)-binding site, which is by nature a common donor for the methyl group (Catoni, 1953, Schlenk and Depalma, 1957). In mammals, the function of several METTL proteins has been characterized, including the formation of N6-methyladenosine (m6A) in mRNA by a complex of METTL3 and METTL14 in human cells (Roundtree et al., 2017, Fu et al., 2014). METTL8, a member of a group of methyltransferase-like proteins, is originally identified as a tension induced protein (TIP) that is involved in mesenchymal to adipose tissue transition (Badri et al., 2008, Jakkaraju et al., 2005); it is also found to be mutated in colon cancers (Yeon et al., 2018) and regulates mouse embryo stem cell differentiation (Gu et al., 2018). Although biochemically METTL8 has been shown to modify m3C in RNAs (Xu et al., 2017), the molecular mechanism(s) regarding METTL8's role in epigenetic-related pathways remain poorly understood.

R-loops are naturally occurring genome-wide structures that are essential for diverse cellular functions (Ginno et al., 2012, Skourti-Stathaki et al., 2011, Yu et al., 2003). Deleterious effects of an excess number of R-loops have been linked to genome instability and DNA replication impairment (Aguilera and Garcia-Muse, 2012, Tuduri et al., 2009), and factors (SETX, TOPI, RNase H, Xrn2) that attenuate R-loop accumulation have been well reported (Aguilera and Garcia-Muse, 2012, Hatchi et al., 2015, Kogoma et al., 1993, Skourti-Stathaki et al., 2011, El Hage et al., 2010, Manzo et al., 2018). Owing to its DNA:RNA hybrid nature, R-loop formation usually occurs at the GC skew promoter regions of a genome (Chen et al., 2017, Ginno et al., 2012, Ginno et al., 2013, Nadel et al., 2015, Sanz et al., 2016, Stork et al., 2016), and transcriptional supercoiling of the DNA also facilitates the formation of R-loops (Gottipati and Helleday, 2009, Brochu et al., 2018, Drolet, 2006, Drolet and Brochu, 2019). In addition to promoters, G-rich sequences have also been identified as transcription pausing elements downstream of poly(A) sites, where they promote poly(A)-dependent transcriptional termination (Gromak et al., 2006) that also involves R-loop formation. The intronic sequence also contributes to alternative splicing by promoting *trans*-splicing (Takahara et al., 2005). Additionally, computational prediction of the G-rich R-Loop Zooms in R-loop DB (Jenjaroenpun et al., 2017) revealed the genome-wide distribution of R-loops, which indicates the possible link between transcription and R-loops, suggesting that the latter may constitute a fundamental structure that impacts transcriptional elongation, termination, and overall outputs. To the best of our knowledge, although R-loops are emerging as “a double-edged sword” to genomic structure (Skourti-Stathaki and Proudfoot, 2014), the only factor that positively regulates R-loops is METTL3 through its m6A activity (Yang et al., 2019) and the biological function of m3C remain unclear.

Here, we unravel the above questions through purification of the METTL8 complex. The overlapping nucleolus localization of METTL8 and R-loops (Hatchi et al., 2015, Salvi and Mekhail, 2015, Skourti-Stathaki et al., 2014), as well as the identification of the METTL8-THOC2-RPA3 association, indicates that regulatory R-loops are maintained in the nucleus, especially in the nucleolus, through METTL8 depending on its m3C activity. Intriguingly, SUMOylation guides METTL8 to the nucleolus, whereby it plays a stabilizing role for the complex via the SUMO-SIM interaction; both the m3C activity and SUMOylation are essential for METTL8 tumorigenicity in xenograft mice models. Taken together, our work establishes a novel R-loop pathway where SUMO-modulated nuclear methyltransferase (METTL8) orchestrates genetic structure R-loops through its enzymatic activity on m3C.

## Results

### Biochemical Revelation of METTL8's Association with R-Loops and RNA Splicing and Export Factors

**Figure 1:**
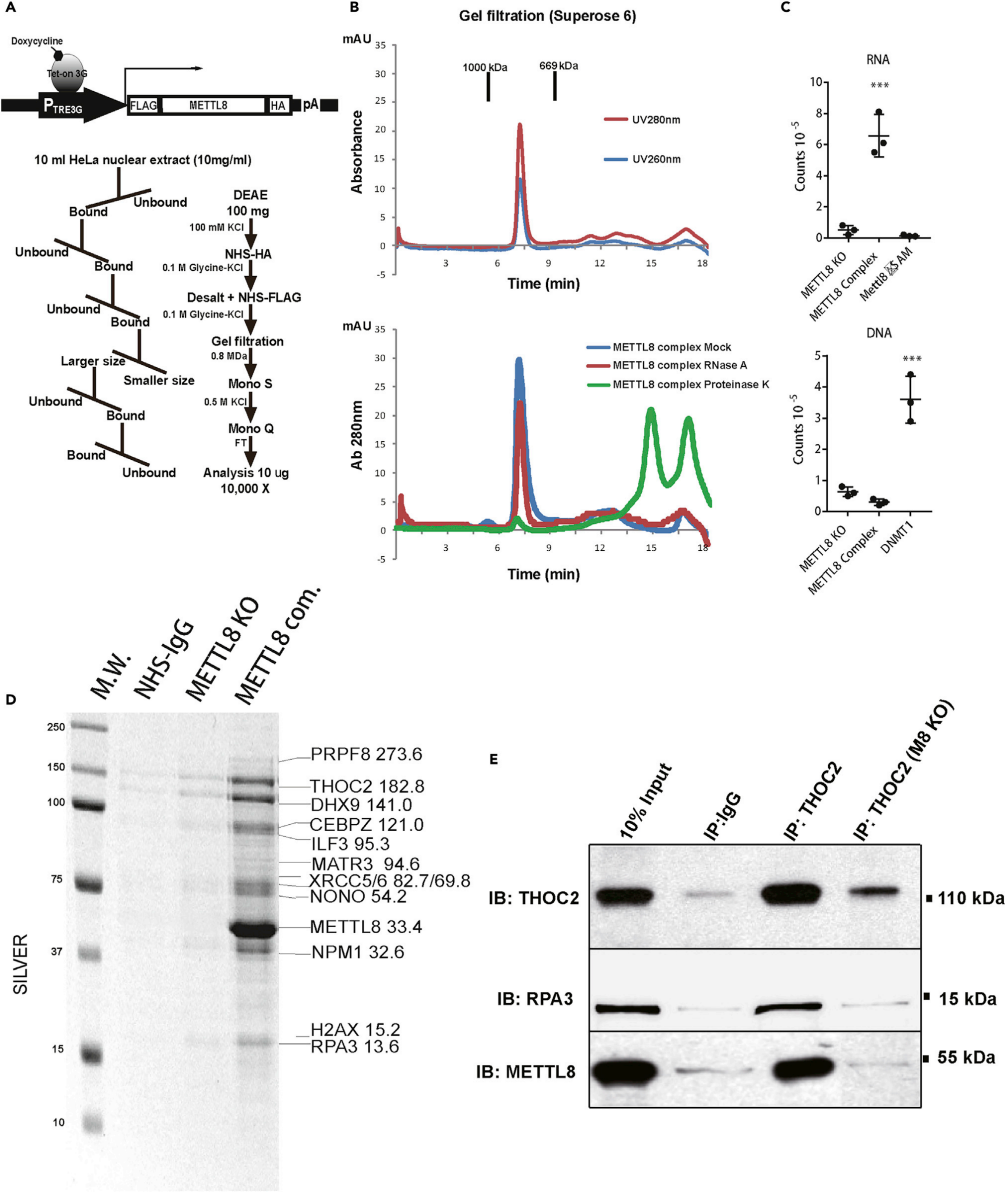
Biochemical Revelation of METTL8's Association with RNA Splicing and Export Factors (A) Schematic representation of the doxycycline-inducible double-tagged (FLAG/HA) expression vector (top). Large-scale (>10 L) METTL8 complex purification steps (bottom). (B) Top panel, gel filtration graph (Superose 6) showing the native size of the nuclear METTL8 complex using dual-channel UV (UV280nm, UV260nm). Bottom panel, gel filtration graph (Superose 6) detected by UV absorbance at 280nm of the METTL8 complex either treated with RNase A (Red) or Proteinase K (Green). (C) Top panel, *in vitro* methylation of RNA by METTL8 complex purified either from wild-type (WT) or METTL8 KO cells; recombinant METTL8 mutant (METTL8ΔSAM, enzymatic dead) were included for negative control. Bottom panel, *in vitro* methylation of DNA by METTL8 complex purified either from WT or METTL8 KO cells; DNA methyltransferase DNMT1 was included for positive control. (D) Silver staining of the purified nuclear METTL8 complex on 12% acrylamide gel; METTL8 KO cells and NHS column conjugated with IgG were included for control. Mass-spectrometry-determined peptide sequences and bands were labeled according to their molecular size. (E) Endogenous coimmunoprecipitation using agarose beads conjugated with anti-THOC2 antibody; METTL8 and RPA3 were shown to interact with THOC2 *in vivo*.

Increasing evidence indicated that nuclear factors play critical roles in epigenetic regulation; we decided to investigate the function of a previously enigmatic epigenetic factor family (METTL2/6/8, Figure S1A). Our approach for the initial study was to use a “guilty by association” strategy (Zheng et al., 2003) to unravel associated partners/potential functions of the larger family member, METTL8, through a series of large-scale conventional (>10 L of mammalian suspension cell line) biochemical purification steps. To ensure the purity of the final complex, a HeLaS3 cell line (ATCC, CCL-2.2) clone that stably expressed inducible FLAG/HA-double-tagged METTL8 (Figure 1A, top) was established. METTL8 expression was induced at the lowest level (Figure S1B, lane 1), and 100 mg of nuclear extract made from this clone was subjected to a scheme of chromatographic steps (Figure 1A, bottom). The complex native size was characterized with Superose 6 analytical gel filtration column to be ∼800 kDa (Figure 1B, top), and it was resistant to RNase A digestion (Figure 1B, bottom, red line), suggesting the METTL8 protein complex is held together through direct interaction. Through the purification process, the METTL8 complex was traced with commercially available monoclonal antibody and its ability to methylate RNAs (Figure 1C, upper) but not DNAs (Figure 1C, down). Preparative size exclusion chromatography (Sephacryl S-300) showed that the nuclear complex is slightly larger than its cytosolic counterpart (Figure S1C), which was suggested to be involved in protein translation regulation through affecting the formation of ribosome-mRNA polysome (Gu et al., 2018). The protein components were visualized on silver staining (Figure 1D, denoted as METTL8 Com.). Their sequence identities were then determined by FASP protein mass spectrometry (see Methods, Table S1). We repeated the same purification scheme (Figure 1A) using NHS columns (GE Healthcare) conjugated with either IgG as a blank control (Figure 1D, NHS-IgG) or METTL8 knockout cells (Figure 1D, METTL8 KO); mass spectrometry analysis of the IgG control was also performed (Table S1) to evaluate background noise.

After repeated mass spectrometry analysis (n = 3), METTL8 was confirmed to be in complex with several well-known protein complexes such as the nucleolar SWAP (NPM1-NCL-PARP1) complex (Borggrefe et al., 1998), the DNAPK (KU70/80, PRKDC, DHX9) complex (Gottlieb and Jackson, 1993), the mammalian homolog of the yeast Tho/Trex complex component THOC2 (a well-established R-loop regulator [Bhatia et al., 2014, Gomez-Gonzalez and Aguilera, 2007, Gomez-Gonzalez and Aguilera, 2009, Gomez-Gonzalez et al., 2011, Gonzalez-Aguilera et al., 2008, Hamperl and Cimprich, 2014, Salas-Armenteros et al., 2017]), and parts of the Spliceosome/U1, U5. 4/6 snRNP complex (summary in Table S1). Key interactions from the Tho/Trex complex were further validated using a THOC2 antibody; endogenous THOC2 was shown to be associated with METTL8 and RPA3 but not with IgG control, and METTL8 knockout (KO) cell lysate was also included for specificity control (Figure 1E). Moreover, evidence from GST pull-down assay indicated purified METTL8 interacted directly with THOC2 and RPA3 (Figure S1E). Thus far, our purification efforts revealed the identity of the METTL8 nuclear complex and elucidated potential functions in which METTL8 may take part (RNA splicing and maturation). Owing to the fact that METTL8's binding partners, TREX (THOC1/2) and RPA family, have well-established roles in R-loop regulation, we are particularly interested in whether METTL8 is a novel factor for this pathway.

### METTL8 Binds to RNA and Regulates R-Loop Formation on the Ribosomal RNA Gene

**Figure 2:**
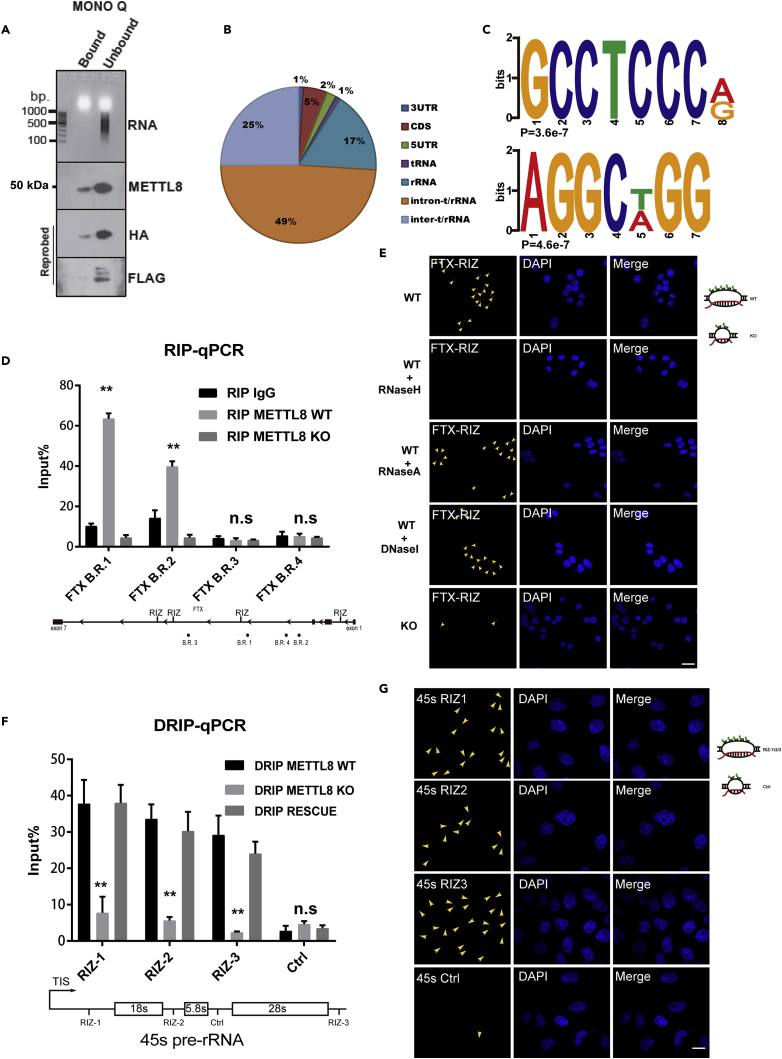
METTL8 Binds to RNA and Regulates R-Loop Formation on the Ribosomal RNA Gene (A) Purified nuclear METTL8 complex from the final step (Mono Q column) (Figure 1A) was collected either for immunoblotting (bottom panels, the original probe is METTL8 [rabbit], then stripped (Figure S2A) and re-probed with HA [rabbit] and FLAG [mouse]) or for RNA extraction; RNA was visualized on an agarose gel (top panel). (B) PAR-CLIP classifications of RNA species binding to the METTL8 complex (complete list in Table S2, control in Table S3). (C) Motif analysis of METTL8-binding RNA that is rich in Gs and Cs (top two motifs, analysis was performed using the DREME online server). (D) Validation of METTL8 interaction with RNA via immunoprecipitation followed by quantitative RT-PCR indicated as RIP-qPCR, the primers were designed around the locus of the METTL8-binding regions (B.R.) detected in PAR-CLIP (schematic at bottom), and amplification folds of the FTX (B)R. (1–4) were normalized to input RNAs; data are means ± SD (n = 3, ∗∗p < 0.01, Student's t test); METTL8 KO cell lysates were included for negative control. (E) FISH confocal imaging indicating that RIZ-FTX (arrowheads) loses (upper right, schematic) its nuclear localization after METTL8 knockout (KO); scale bar, 10 μm; treatment with RNase H, RNase A, and DNase I was included as controls. (F) DNA:RNA hybrid immunoprecipitation (DRIP) analysis of HeLa METTL8 WT and KO cells; the number of R-loop-binding sites (on 45s pre-rRNA) was calibrated against the input nucleic acids; data are means ± SD (n = 3); ∗∗p < 0.01, Student's t test. Bottom boxes, a schematic of 45s pre-rRNA with the location of RIZ-1/2/3 indicated; a gene site without RIZ was included as control (Ctrl). (G) Fluorescence *In Situ* Hybridization (FISH) confocal microscope imaging using the RIZ probes (green) indicated in (F, RIZ-1/2/3). Arrowheads indicate R-loops detected by the probes and a schematic figure was shown on the upper right.

During biochemical analysis of the complex, we observed OD260/OD280 > 2 at the peak center of METTL8 complex (Figure 1B), which suggested that the complex is not entirely composed of protein and that the other components are likely to be RNAs (Glasel, 1995). Our first approach in confirming the binding of RNAs is Trizol (Invitrogen) phase separation of the purified complex. We followed the commercially available extraction protocol, and the RNAs were extracted and visualized on a gel in conjunction with the Trizol-denatured HA/FLAG-tagged METTL8 protein (Figure 2A); the HA/FLAG blot was stripped/reprobed from the original METTL8 blot (Figure S2A). Next, the HA/FLAG-METTL8 over-expressing cells were subjected to PAR-CLIP (see Methods) sequencing, and a total of 1,252 unique RNAs (with an enrichment factor >2) were identified (Table S2). The bound RNAs were mostly t/r-RNA introns (49%) and inter t/r-RNA (25%) in the nucleolus (Figure 2B). Moreover, the METTL8-binding RNA motif was determined to be GC rich using DREME (Bailey, 2011) (Figure 2C, top two hits with p = 3.6 × 10^−7^ and p = 4.6 × 10^−7^). We also sequenced background-binding RNAs to the FLAG antibody in METTL8 KO cells; our data have indicated that they are mostly ribosomal and skeleton (microtubule) gene RNAs (Table S3). In addition to our earlier evidence regarding its binding with the R-loop regulator, TREX (THOC1/2) (Figures 1D, 1E, and S1E), we reason that METTL8 complex might be involved in R-loop regulation, as R-loops are reported to be GC skewed (Chen et al., 2017, Jenjaroenpun et al., 2017).

We validated one of the top PAR-CLIP hits using RNA immunoprecipitation (RIP-qPCR); the result indicated that five prime to Xist (FTX), which was reported to affect X-inactivation, binds exclusively to METTL8 complex in binding region 1 (B.R. 1) and B.R. 2 but weakly to B.R. 3/4;, METTL8 KO cells were included for negative control (Figure 2D, schematic of the B.R. genome location at the bottom). METTL8 was knocked out from HeLa cells using CRISPR-Cas9 targeting exon 3 (Figure S1D), and off-targets were checked *in silico*. FISH (fluorescence *in situ* hybridization) imaging (Napoli, 2013) using FTX probes for the R-loop-prone region (online-computed GC-rich probes [Jenjaroenpun et al., 2015], denoted as R-loop Initiation Zooms, RIZs) showed more displaced GC-rich single-stranded DNA in the nucleolus of WT HeLa cells than in KO METTL8 (Figure 2E top and bottom panels, Schematic Figure on right); treatment with an RNA:DNA hybrid-specific enzyme (RNase H) abolishes the FISH signal, whereas RNase A and DNase I have insignificant effect (Figure 2E middle panels, quantification in Figure S2E, n = 4, ∗∗∗p < 0.005).

Regulation of R-loops by METTL8 was evaluated on the 45s pre-rRNA gene using DNA:RNA hybrid immunoprecipitation–quantitative PCR (see Methods, DRIP-qPCR). DRIP-qPCR indicated that the R-loops from RIZ-1, RIZ-2, and RIZ-3 sites were significantly (n = 5, ∗∗p < 0.01) reduced in the METTL8 KO samples (Figure 2F, schematic on bottom). The control (Ctrl) site without RIZ sequence does not bind to the S9.6 antibody and remains unchanged after METTL8 KO, suggesting METTL8 directly affects R-loop formation on selected gene loci that are mostly (49%) t/r-RNAs (Figure 2F). FISH imaging (Napoli, 2013) indicated more R-loops at 45s RIZ-1, RIZ-2, and RIZ-3 than in the control region (Figure 2G), and quantification of R-loop positive cells was deemed significant (Figure S2F, n = 4, ∗∗p < 0.001, ∗∗∗p < 0.005, ∗∗∗∗p < 0.0001), suggesting that these R-loops are formed selectively. With evidence from both its R-loop-related co-factors (THOC2 and RPA3), together with indications from its binding to GC-skew RNAs that is sensitive to RNase H and the DRIP-qPCR data, and repeated validations on other models genes such as the *ACTB* gene (Figures S2B–S2D), we reasoned that METTL8's function is centered in R-loop regions. Therefore, we developed assays to investigate this hypothesis.

### METTL8 Induces R-Loops in Cell Nucleolus

**Figure 3:**
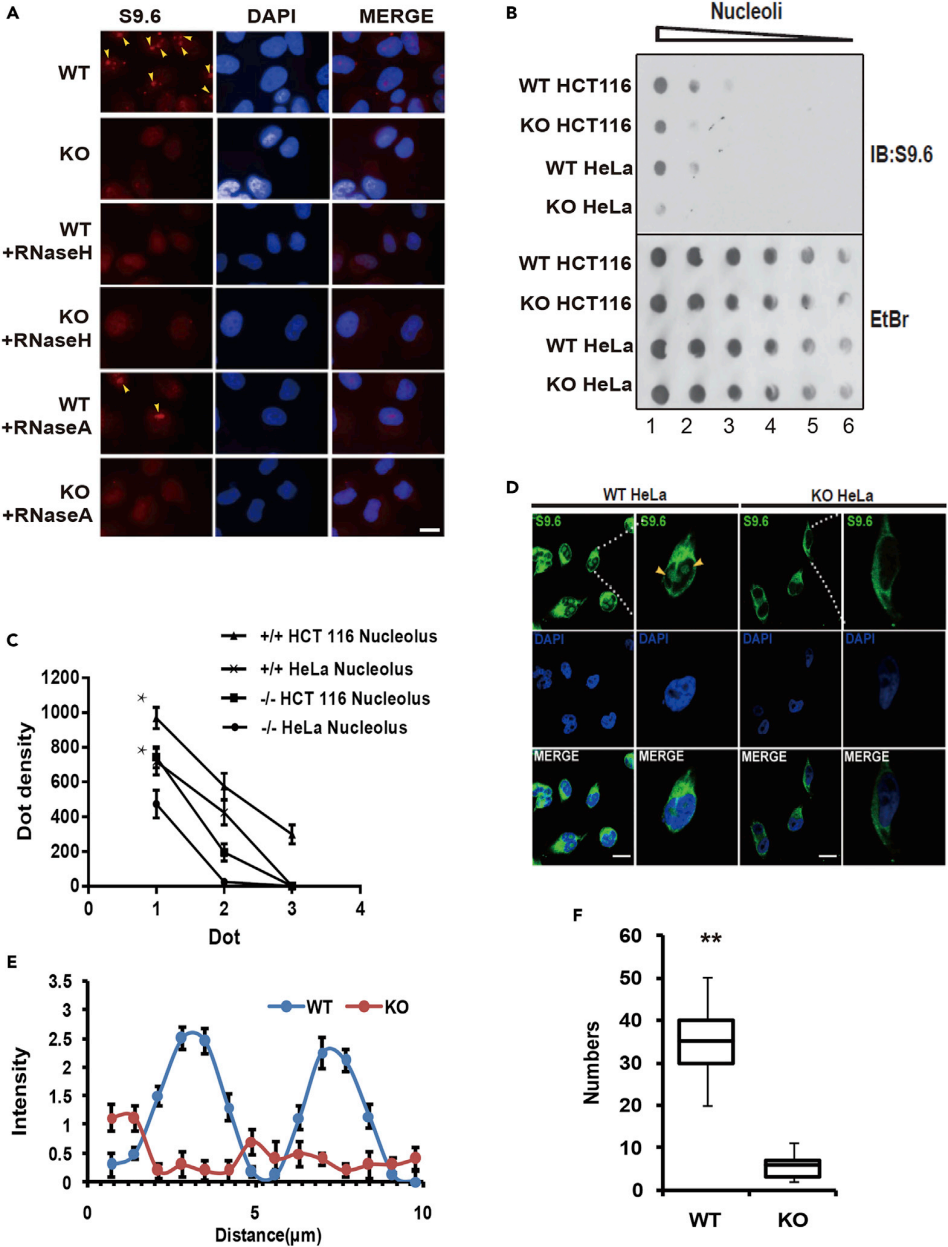
METTL8 Induces R-Loops in Cell Nucleolus (A) R-loops (red) immunofluorescence imaging of WT and KO HeLa nuclei either mock treated or treated with RNaseH (1 μg/mL) and RNase A (1 μg/mL). Scale bar, 10 μm. (B) Dot blot displays of R-loop structures from the nucleolar extract. Loading was visualized by ethidium bromide (EtBr) staining; the number of R-loops was visualized with the S9.6 antibody (IB: S9.6). (C) Dot blot densitometry analysis of HeLa and HCT116 nucleolar R-loops from repeated assays (n = 3, ∗p < 0.05, Student's t test). (D–F) The representative confocal image (D) of R-loops with an S9.6 antibody; R-loops (green) were stained with an S9.6 antibody, and DAPI (blue) was included to counter-stain the HeLa nucleus. Scale bar, 10 μm. Line scan analysis (E) of WT (blue) and KO (red) HeLa cells (means ± SD, n = 3). Statistical analysis (F) of R-loop-positive cells; data are means ± SD (n = 204). ∗∗p < 0.01, Student's t test.

To quantify the number of R-loops *in vitro*, we customized a dot blot assay by serial dilution of equal amounts of starting nucleic acids on a nylon membrane (positively charged, Roche). The loading quantity was checked with EtBr (ethidium bromide). Initially, the detected number of R-loops from the total nucleic acid extract was similar between METTL8 WT and KO cell lines (Figure S3A); however, after repeated (n > 3) investigation supported by a recent publication (Zhang et al., 2015), we found that there are cross-reactions of the anti-DNA-RNA hybrid (S9.6) antibody with AU-rich RNA:RNA duplexes that are abundant in the cytosol. Therefore, the pure cell nucleus and/or nucleolus was isolated (Figure S3B), and a biochemical marker of each cellular compartment was checked with antibodies against UBF1 (a nuclear marker) NCL (nucleolus marker) or alpha-tubulin (a cytoplasm marker) (Figure S3C). The specificity of the S9.6 antibody was monitored by comparing the purified nuclei (fixed on a slide) R-loop signals before and after RNase H and RNase A treatment, which digest R-loops and single-stranded RNAs, respectively (Figure 3A). The results in Figure 3A clearly indicated that the S9.6 antibody specifically labels the nuclear R-loops and no cytoplasmic signals were detected after nuclear isolation.

After having developed a reliable R-loop quantification method using pure nucleoli, the difference in the number of R-loops between WT and KO cell nucleoli were quantified using dot blots (Figure 3B, lane 1–6). The dot intensity analysis (quantified by ImageJ) indicates that the number of R-loops from METTL8 KO cells was significantly lower (Figure 3B, rows 2 and 4) from repeated assays (Figure 3C, n = 3, ∗p < 0.05). The above finding was also confirmed multiple times by confocal immunofluorescence imaging (Figures 3D–3F and S3D). We showed that the nucleolus R-loop signal, in many cases, completely diminished after Mettl8 ablation (Figure 3D, enlarged inset), whereas the cytoplasmic signal remained detectable (cytoplasmic AU-rich RNAs). The line intensity graph (quantified by ImageJ line intensity plugin) indicated a nuclear localization of R-loops in WT but not KO cells (Figure 3E, n = 3). Up to 10 unbiased image fields (Figure S3D) were taken, and the R-loop difference between WT and KO HeLa S3 cells was determined to be significant (Figure 3F, n = 204, ∗∗p < 0.01).

### Nuclear Localization of METTL8 Is Maintained by SUMOylation at Lysine 80

**Figure 4:**
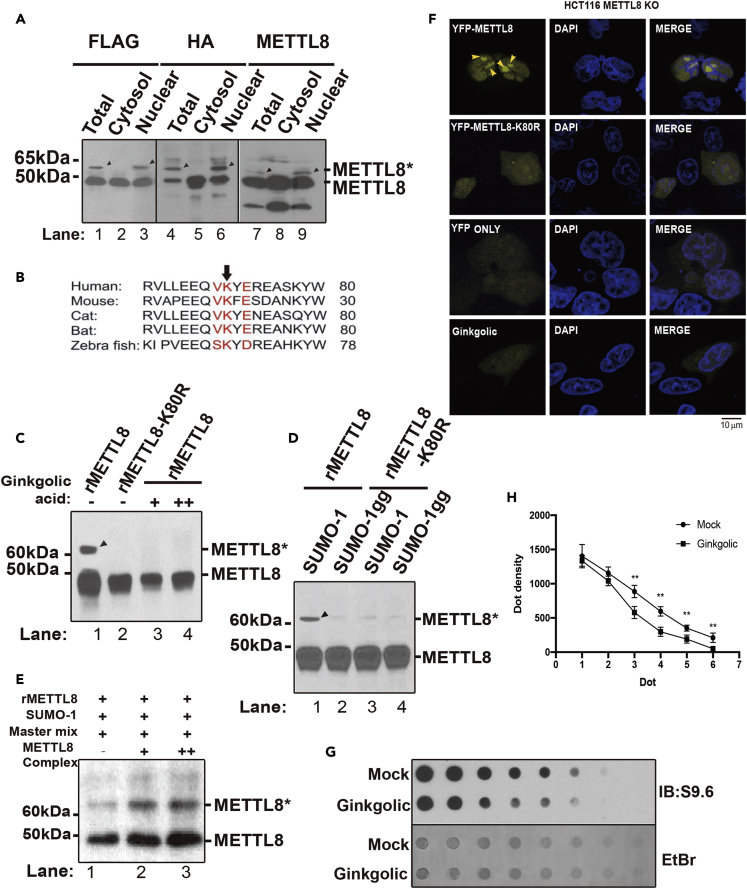
Nuclear Localization of METTL8 Is Maintained by SUMOylation at Lysine 80 (A) Western blot analysis of METTL8 protein band patterns (modifications) in fractioned or whole HeLa cell lysates with three different antibodies; bands were visualized separately in parallel using FLAG, HA, and METTL8 antibodies (left to right); the molecular weight is indicated on the left. (B) Motif analysis of METTL8 protein at its lysine (K) SUMOylation site across species. (C–E) *In vitro* SUMOylation of recombinant METTL8 (rMETTL8) protein. (C–E) (C) Together with the SUMOylation Master Mix, the bacterial purified rMETTL8 or SUMOylation (rMETTL8-K80R) mutant protein was either incubated with two different analogs of the SUMO protein (SUMO-1, SUMO-1gg [inactive]), (D) an increasing dose of SUMO E3-ligase inhibitor (Ginkgolic acid), (E) or with an increasing quantity of purified METTL8 nuclear complex (from Figure 1D). The molecular weight of SUMOylated METTL8 protein is denoted on the left, and the modified band is denoted as METTL8∗ on the right. (F) Confocal imaging of either YFP-METTL8 or YFP-METTL8-K80R plasmids transfected into METTL8 KO HCT116 cells (yellow arrowheads), YFP and YFP-METTL8 transfected cells treated with Ginkgolic acid were included as controls, nuclei were counter-stained with DAPI (blue); scale bar, 10 μm. (G) Dot blot of R-loop structures from nucleolar extracts with or without Ginkgolic treatment (10 μg/mL); loading and R-loop structure was visualized with EtBr and the S9.6 antibody (IB: S9.6), respectively. (H) Dot blot densitometry analysis of (G), n = 3, ∗∗p < 0.01, Student's t test.

One interesting observation arising from complex purification is that extra bands appeared in the nuclear fraction of METTL8 but not from the cytosolic fraction (Figure S1C). This prompted us to test whether the METTL8 protein is post-translationally modified. We confirmed the molecular shift to be approximately 10 kDa by incubating cell lysates with three antibodies (anti-FLAG, anti-HA, and anti-METTL8) targeting the same double-tagged METTL8 protein (Figure 4A). Analysis of the amino acid motif suggested this modification is SUMO on a conserved lysine across species (Figure 4B, lysine 80 in humans). Next, *in vitro* SUMO assays using purified recombinant proteins (rMETTL8, rMETTL8-K80R, SUMO-1, and SUMO-1gg [lacking C terminus]) indicated that our prediction is correct. Point mutation of the SUMO receptor lysine (K) to arginine (R) completely prevented METTL8 SUMOylation (arrowhead) *in vitro* (Figure 4C, lanes 3 and 4). Additionally, an increased amount of Ginkgolic acid, a known SUMO ligase inhibitor, completely abolished the METTL8 SUMOylation (arrowhead) *in vitro* (Figure 4D, lanes 3 and 4). We also showed that addition of purified METTL8 complex promotes the *in vitro* SUMOylation reaction, suggesting SUMOylatin of METTL8 is independent from the complex but is enhanced when the complex is formed (Figure 4E). Computational analysis indicates that METTL8 is the only member of the METTL2/6/8 family that contains both a SUMO site and a SUMO interaction motif (SIM) (Table S4). YFP fusion protein (YFP-METTL8, YFP-METTL8-K80R) imaging suggests that lysine 80 is responsible for METTL8's nuclear enrichment in METTL8 KO HCT116 cells (Figure 4F, arrowheads; Figure S3E, showing the KO effect), and Ginkgolic acid inhibits not only the nuclear enrichment of METTL8 (Figure 4F, bottom panels) but also its downstream genomic structure: R-loops (Figures 4G and 4H). We conclude from the above evidences that METTL8 is SUMOylated at a conserved site (lysine 80 in human), which is essential for its function on R-loops in the nuclear compartment. Next, we proceed to explore the biological connections between these motifs (SAM and SUMO) with R-loops.

### SAM Domain Is Essential for METTL8 R-Loop-Inducing Ability via m3C

**Figure 5:**
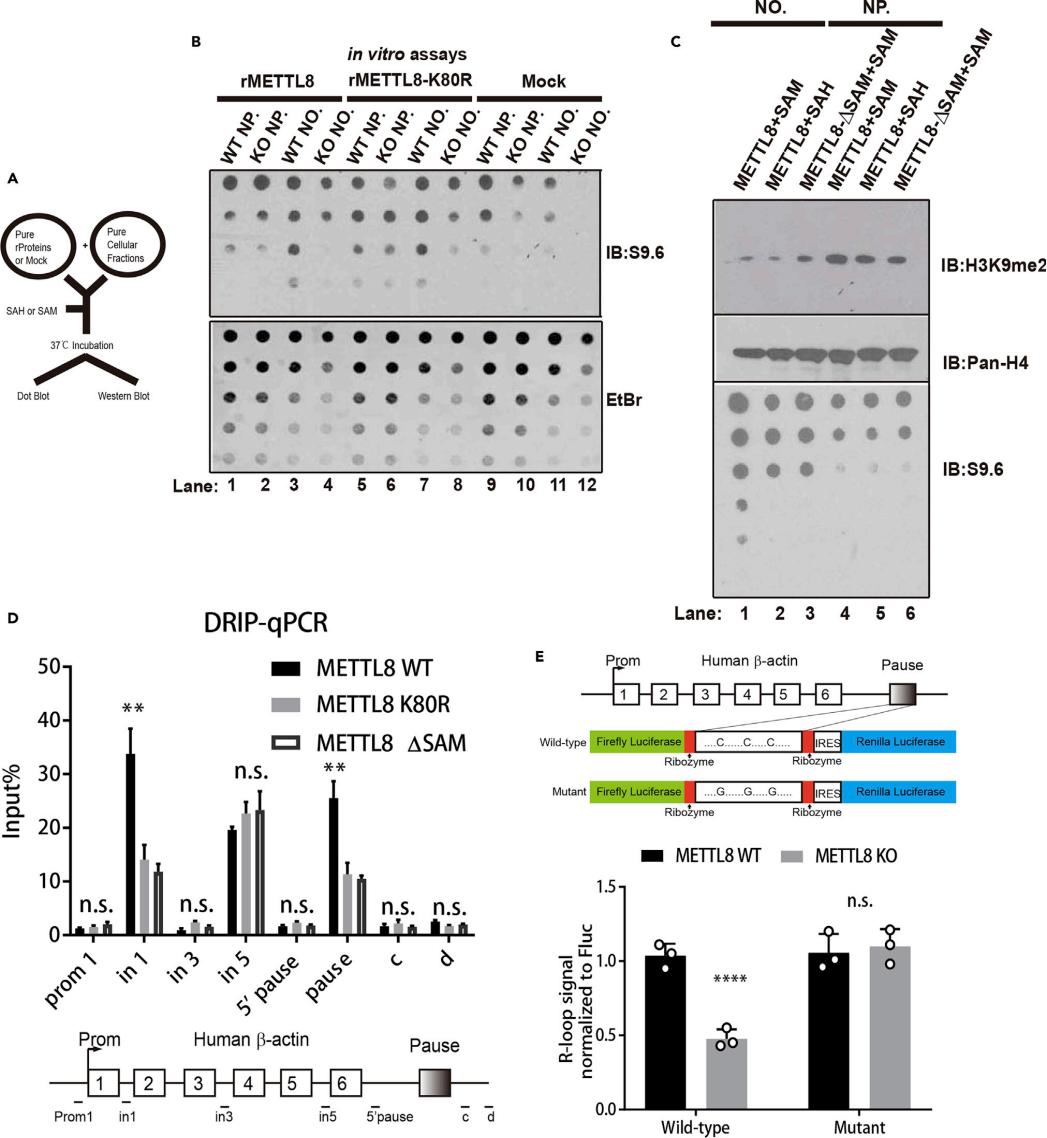
SAM Domain Is Essential for METTL8 R-Loop-Inducing Ability via m3C (A) Schematic of the *in vitro* methylation assay; components were mixed as indicated and incubated at 37°C followed by either a dot blot or western blot. (B) *In vitro* methylation of genomic nucleic acid with purified recombinant proteins, compared with a control (lane 12), rMETTL8-K80R retained its R-loop-inducing ability in KO nucleolar (NO.) (lane 8). (C) *In vitro* methylation of nucleolar (NO.) and nucleoplasm (NP.) nucleic acids with recombinant proteins; wild-type METTL8 is able to increase R-loops in NO. (lane 1) but not in NP. (lane 4); the methyl-donor mutant (METTL8-ΔSAM) also fails to increase R-loops in NO.(lane 3). (D) DNA:RNA hybrid immunoprecipitation (DRIP) analysis of METTL8 WT, METTL8 K80R, and METTL8 ΔSAM inducing R-loops in HeLa cells; the number of R-loop-binding sites (on beta-actin) was calibrated against the input nucleic acids; data are means ± SD (n = 3); ∗∗p < 0.01, Student's t test. Bottom boxes, a schematic of human beta-actin gene dash lines indicating Prom1, in1, in3, in5, 5'pause, and the pause site. (E) Top, schematic of the luciferase reporter system for R-loop-induced transcription pause. The *ACTB* gene pause region, containing m3C sites (Cs) or mutated sites (Gs), was inserted into a dual luciferase reporter vector. Bottom, quantification of R-loops and readthrough products of the wild-type and mutant reporters in METTL8 WT and METTL8 KO cells. Data are means ± SD (n = 3, ∗∗∗∗p < 0.0001, Student's t test).

*In vitro* assays (Figure 5A, schematic) indicated that, although the K80R METTL8 mutant loses its nuclear localization, it retained its ability to induce R-loops *in vitro*, suggesting that SUMO only affects its cellular localization but not its enzyme activity toward R-loops (Figure 5B, lane 8 versus lane 12). A possible explanation for this observation is that SUMOylation frequently targets entire groups of physically interacting proteins and serves as a distinguishing marker for functionally engaged protein complexes (Jentsch and Psakhye, 2013). Additionally, METTL8 is the only member of its family that contains both a SUMO and a SIM motif (Table S4); therefore, we conclude that the METTL8 complex is an active, functional nuclear complex cross-linked by SUMO-SIM interaction. The enzymatically dead counterpart (rMETTL8-ΔSAM, lacking the canonical S-Adenosyl Methionine donor domain) fails to induce R-loops (Figure 5C, lane 3 versus lane 1, Pan-H4 for loading control), which indicates the SAM domain (methyl donor) is responsible for its R-loop stabilizing activity. We also tested the R-loop inducing ability of these METTL8 mutants; DRIP-qPCR has indicated that both the K80R and ΔSAM mutant failed in inducing R-loops on “in 1” and “pause” sites, whereas WT METTL8 could (Figure 5D), suggesting that, although K80R could induce R-loops *in vitro* (Figure 5B), the dislocation of the K80R mutant abolishes its ability to induce R-loops in cells (also supported by its inhibitor in Figure 4G). To investigate the effect of m3C on R-loops stability, we constructed a minigene reporter system in which two luciferases (Firefly and Renilla, Figure 5E, top) are separated by the *ACTB* pause element that contains C-rich RIZ. R-loop formation would induce transcription pause that only produces the Firefly transcripts, whereas reduction of R-loop would generate readthrough, which in turn produces both the Firefly and the Renilla transcripts. Our data showed that METTL8 KO causes a reduction in R-loops only in the wild-type luciferase reporter but not in the Mutant reporter where Cs has been mutated to Gs (Figure 5E, bottom), suggesting METTL8 regulates R-loop-induced transcription pause via its ability to modify m3C. Having validated various mechanistic aspects of METTL8 on R-loop biogenesis from Figure 1, Figure 2, Figure 3, Figure 4, Figure 5, taking into consideration that earlier report indicated R-loops affect genome stability and are potentially tumorigenic (Hamperl et al., 2017). We proceeded to test the tumorigenicity of METTL8 using xenograft mice models.

### The SAM and SUMO Domains Are Tumorigenic in Mice Model

**Figure 6:**
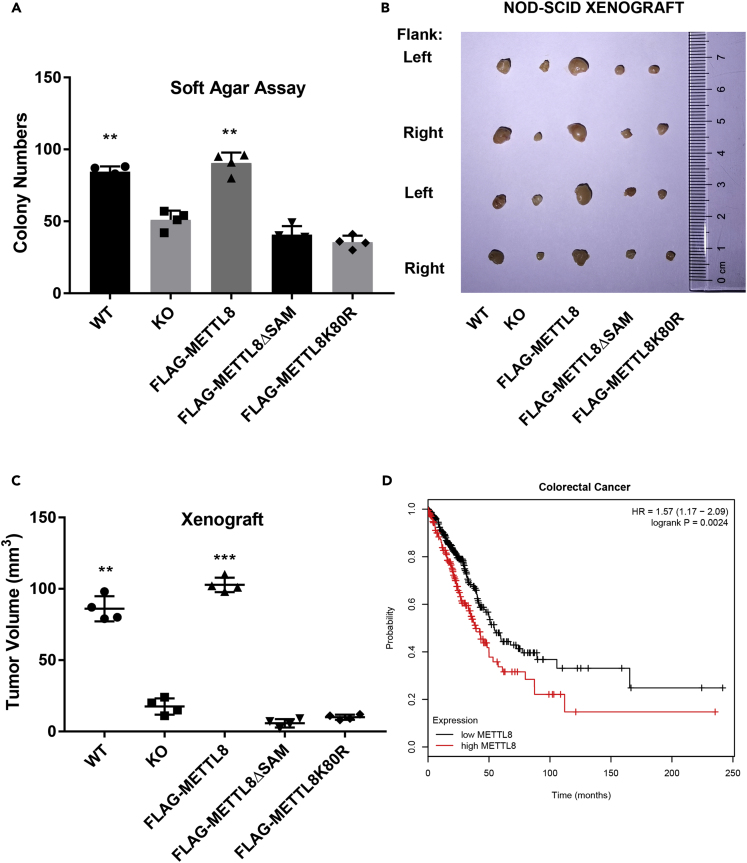
The SAM and SUMO Domains Are Tumorigenic in Mice Model (A) HCT116 WT or KO cells overexpressing METTL8, ΔSAM, and K80R were seeded at 2,000 cells/well in quadruples, and after 2 weeks, colonies grown up in soft agar were apparent. Colonies with more than 50 cells were counted, data are mean ± SD (n = 4, ∗∗p < 0.01, Student's t test). (B and C) 1 × 10^5^ HCT116 WT or KO cells overexpressing METTL8, ΔSAM, and K80R were mixed with matrigel and injected subcutaneously into the left or right flank on the back of 6-week-old female NOD-SCID mice; after 4 weeks, tumor tissue was dissected, photographed (B), and weighted (C, n = 4, ∗∗p < 0.01, ∗∗∗p < 0.005, Student's t test). (D) Patients with colorectal cancer as enlisted in the study (GSE17536&37) were stratified according to METTL8 expression level, and survival rate was monitored in a cohort of patients (n = 504, p = 0.0024, log rank test) with the indicated Mettl8 gene level.

In Figure 3 we have showed METTL8 promotes R-loops formation in cell lines, and since R-loops are reported to be tumorigenic, we proceeded to test if METTL8 could promote tumorigenesis via regulating R-loops. First, we showed in soft agar colony assay, METTL8 promotes cell proliferation and both its m3C donor domain and SUMOylation domain are essential for this function (Figure 6A). Next we showed in immunodeficient mice xenograft models that METTL8 KO HCT116 cells (Figure S3E) and their mutants, METTL8ΔSAM and METTL8K80R, are less tumorigenic than their wild-type counterpart (Figure 6B) and their difference in tumor volume is deemed to be significant (Figure 6C, n = 4, ∗∗p < 0.01, ∗∗∗p < 0.005). Xenograft data suggested that both the SAM and SUMO domains, which were shown to affect METTL8 R-loop-inducing ability (Figure 5), are essential for the tumorigenicity of METTL8. Clinically, based on published datasets in human patients with colorectal cancer (Smith et al., 2010), high level of METTL8 significantly has a lower survival rate than the low-level cohort (Figure 6D, n = 504, p = 0.0024), indicating METTL8 may contribute to therapeutic response in patients with cancer.

Taken together, our data showed METTL8 regulates R-loops through its methyltransferase activity, which was identified to be m3C (Xu et al., 2017), and its SUMOylation on Lysine 80 contributes to its nuclear enrichment, which is essential for its R-loop inducing activity. Xenograft mice models also suggested that both the SAM and the SUMO domains are responsible for METTL8 tumorigenicity. The METTL family is emerging to be an interesting class of proteins that regulate various aspects of RNA metabolism such as circRNA (Zhou et al., 2017) and R-loop (Yang et al., 2019), which are both related to tumorigenesis and genome stability.

## Discussion

### METTL8 Complex and R-Loops

Epigenetic regulation is generally recognized to be related to covalent modifications of histones within chromatin or of genetic material such as DNA itself (Allfrey et al., 1964, Gold et al., 1963a, Gold et al., 1963b), which essentially impacts the transcriptional landscape. Recent research has found the prevalent, non-canonical genomic structure of R-loops, which are associated with epigenetic signatures in mammals (Sanz et al., 2016); however, the mechanisms by which R-loops regulate genetic readout (transcription or RNA processing) and detail information regarding the role of R-loops in RNA splicing remain elusive. Here, through biochemical approaches, we first show that the METTL8 complex is an RNA processing complex containing well-characterized proteins involved in RNA splicing, mRNA export, and spliceosome assembly (Figure 1). We show that its key component, METTL8, controls R-loop production in the cell nucleus and, specifically, in the nucleolus (Figure 3), thus physically linking these biological processes together (Aguilera and Gomez-Gonzalez, 2008, Li and Manley, 2006). Interestingly, in addition to the TREX (THOC1/THOC2) complex, the NFAT (IL2/IL3) complex, another R-loop-related METTL8 complex component, Replication Protein A (RPA), has also been identified (Figure 1). RPA is a DNA-binding protein that binds to displaced single-stranded DNA in long and persistent R-loops (>1 kb) in the regions involving class switching (Nguyen et al., 2017, Yu et al., 2003); its co-appearance suggests that METTL8's main function is related to regulatory R-loops in the genome.

### SUMOylation as R-Loop Regulator

We have shown that the SUMOylation signaling pathway regulates METTL8 nucleus-cytoplasm shuttling through an evolutionary well-conserved site (K80 in humans) (Figure 5B). This modification is important for METTL8's enrichment in the nucleus (Figure 4), and it may be critical for the formation of the 800 kDa METTL8 complex; studies have shown that SUMO-SIM (SUMO Interacting Motif) fosters both intra- and inter-molecular interactions in protein complexes (Jentsch and Psakhye, 2013), and METTL8 is the only member of its family that contains both a SUMO and a SIM site (Table S4), which suggests that METTL8 may interact with itself and acts as glue to hold the complex together. In addition to METTL8, other R-loop regulators, such as DNA topoisomerase I (TOP1) and RPA, are also SUMOylated (Li et al., 2015, Jentsch and Psakhye, 2013), indicating that SUMOylation could be an important layer of regulation for R-loop biogenesis. Moreover, SUMO ligases have been shown to commonly target assembled complexes that are functionally engaged (Psakhye and Jentsch, 2012, Dou et al., 2010), and most of these SUMO complexes are found in the nucleus; the nucleolar-enriched METTL8 complex regulating R-loops characterized in this study is another good example of SUMO signaling target.

### Non-Canonical Genetic Structures and Their Regulators

GC-rich non-canonical genetic structures, such as R-loops, circRNAs, G-quadruplex (guanidine-rich) (Parkinson et al., 2002), and I-motifs (cytosine-rich) (Phan et al., 2000), were previously considered mistakes during cell biogenesis. However, more evidences indicate that the GC-rich region of the genome is in fact an important part of the genetic code. Here, we show that the METTL8 complex influences R-loops formation in the nucleus (Figure 3) and it affects R-loop regulated transcription through m3C (Figure 5). It is known that R-loops influence transcription events such as pausing and splicing, together with recent work showing METTL3 could promote R-loop formation and facilitate transcription termination via m6A (Yang et al., 2019), we believe it is likely that nucleic acid base modifications such as methylation could be a generic biologic mechanism in which R-loops are regulated. In addition, our unpublished data have suggested METTL8 could perturb circular RNA production, which is likely due to the regulatory upstream factors such as genomic R-loops.

### Limitations of the Study

Although all data are safely reproduced in this study, some limitations should be noted. In current field, studies on R-loops heavily rely on the S9.6 antibody; assays such as the commonly used DRIP, cell imaging, and quantitative assays (dot blots) are influenced, sometimes distorted, by nonspecific binding to the AU-rich RNAs. We are able to circumvent interference from cytosolic AU-rich RNA by cellular fractioning; we are unable, unfortunately, to assess the influence of nuclear AU-rich RNAs on R-loops detection sensitivity with the current measure.

## Methods

All methods can be found in the accompanying Transparent Methods supplemental file.

## Data and Code Availability

All data are available in the main text or the Supplemental Information.

## Supplemental Information

Document S1. Transparent Methods and Figures S1–S3

Table S1. List of METTL8-Binding Proteins in Known Complexes, Related to Figure 1 List of proteins co-purified with METTL8, including the SWAP complex, the DNAPK complex, the NFAT complex, part of the spliceosome complex, part of the TREX complex, and part of the U5 snRNP complex that were co-purified with METTL8. Mass spectrometry on IgG control was also included for mock control.

Table S2. List of METTL8-Binding RNAs, Related to Figure 2 List of RNAs that bind to METTL8. ncRNA473, FTX, HFM1, and Ankyrin 11 were the top four RNAs among 1,252 RNAs identified. The binding region (B.R.) was listed according to hg19 database.

Table S3. List of RNAs Captured in METTL8 KO Cells, Related to Figure 2 List of RNAs captured by FLAG antibody in METTL8 KO cells.

Table S4. SUMO Site and SUMO Interaction Motif (SIM) in the METTL8 Family, Related to Figure 4 SUMOylation sites were predicted using primary protein sequence. METTL8 contains both a SUMO site at lysine 80 and SUMO interaction site at 114–118. METTL2A/B contains only a SUMO interaction site, and METTL6 contains neither site.

Table S5. List of Primers Used in This Study, Related to Figures 2 and 5

## References

[bib1] Aguilera A., Garcia-Muse T. (2012). R loops: from transcription by-products to threats to genome stability. Mol. Cell.

[bib2] Aguilera A., Gomez-Gonzalez B. (2008). Genome instability: a mechanistic view of its causes and consequences. Nat. Rev. Genet..

[bib3] Allfrey V.G., Faulkner R., Mirsky A.E. (1964). Acetylation and methylation of histones and their possible role in the regulation of RNA synthesis. Proc. Natl. Acad. Sci. U S A.

[bib4] Badri K.R., Zhou Y., Dhru U., Aramgam S., Schuger L. (2008). Effects of the SANT domain of tension-induced/inhibited proteins (TIPs), novel partners of the histone acetyltransferase p300, on p300 activity and TIP-6-induced adipogenesis. Mol. Cell Biol..

[bib5] Bailey T.L. (2011). DREME: motif discovery in transcription factor ChIP-seq data. Bioinformatics.

[bib6] Bhatia V., Barroso S.I., Garcia-Rubio M.L., Tumini E., Herrera-Moyano E., Aguilera A. (2014). BRCA2 prevents R-loop accumulation and associates with TREX-2 mRNA export factor PCID2. Nature.

[bib7] Borggrefe T., Wabl M., Akhmedov A.T., Jessberger R. (1998). A B-cell-specific DNA recombination complex. J. Biol. Chem..

[bib8] Brochu J., Vlachos-Breton E., Sutherland S., Martel M., Drolet M. (2018). Topoisomerases I and III inhibit R-loop formation to prevent unregulated replication in the chromosomal Ter region of *Escherichia coli*. PLoS Genet..

[bib9] Catoni G.L. (1953). S-Adenosylmethionine; a new intermediate formed enzymatically from L-methionine and adenosinetriphosphate. J. Biol. Chem..

[bib10] Chen L., Chen J.Y., Zhang X., Gu Y., Xiao R., Shao C., Tang P., Qian H., Luo D., Li H. (2017). R-ChIP using inactive RNase H reveals dynamic coupling of R-loops with transcriptional pausing at gene promoters. Mol. Cell.

[bib11] Dou H., Huang C., Singh M., Carpenter P.B., Yeh E.T. (2010). Regulation of DNA repair through deSUMOylation and SUMOylation of replication protein A complex. Mol. Cell.

[bib12] Drolet M. (2006). Growth inhibition mediated by excess negative supercoiling: the interplay between transcription elongation, R-loop formation and DNA topology. Mol. Microbiol..

[bib13] Drolet M., Brochu J. (2019). R-loop-dependent replication and genomic instability in bacteria. DNA Repair (Amst)..

[bib14] El Hage A., French S.L., Beyer A.L., Tollervey D. (2010). Loss of Topoisomerase I leads to R-loop-mediated transcriptional blocks during ribosomal RNA synthesis. Genes Dev..

[bib15] Fu Y., Dominissini D., Rechavi G., He C. (2014). Gene expression regulation mediated through reversible m(6)A RNA methylation. Nat. Rev. Genet..

[bib16] Ginno P.A., Lim Y.W., Lott P.L., Korf I., Chedin F. (2013). GC skew at the 5' and 3' ends of human genes links R-loop formation to epigenetic regulation and transcription termination. Genome Res..

[bib17] Ginno P.A., Lott P.L., Christensen H.C., Korf I., Chedin F. (2012). R-loop formation is a distinctive characteristic of unmethylated human CpG island promoters. Mol. Cell.

[bib18] Glasel J.A. (1995). Validity of nucleic acid purities monitored by 260nm/280nm absorbance ratios. Biotechniques.

[bib19] Gold M., Hurwitz J., Anders M. (1963). The enzymatic methylation of RNA and DNA. Biochem. Biophys. Res. Commun..

[bib20] Gold M., Hurwitz J., Anders M. (1963). The enzymatic methylation of rna and DNA, Ii. On the species specificity of the methylation enzymes. Proc. Natl. Acad. Sci. U S A.

[bib21] Gomez-Gonzalez B., Aguilera A. (2007). Activation-induced cytidine deaminase action is strongly stimulated by mutations of the THO complex. Proc. Natl. Acad. Sci. U S A.

[bib22] Gomez-Gonzalez B., Aguilera A. (2009). R-loops do not accumulate in transcription-defective hpr1-101 mutants: implications for the functional role of THO/TREX. Nucleic Acids Res..

[bib23] Gomez-Gonzalez B., Garcia-Rubio M., Bermejo R., Gaillard H., Shirahige K., Marin A., Foiani M., Aguilera A. (2011). Genome-wide function of THO/TREX in active genes prevents R-loop-dependent replication obstacles. EMBO J..

[bib24] Gonzalez-Aguilera C., Tous C., Gomez-Gonzalez B., Huertas P., Luna R., Aguilera A. (2008). The THP1-SAC3-SUS1-CDC31 complex works in transcription elongation-mRNA export preventing RNA-mediated genome instability. Mol. Biol. Cell.

[bib25] Gottipati P., Helleday T. (2009). Transcription-associated recombination in eukaryotes: link between transcription, replication and recombination. Mutagenesis.

[bib26] Gottlieb T.M., Jackson S.P. (1993). The DNA-dependent protein kinase: requirement for DNA ends and association with Ku antigen. Cell.

[bib27] Gromak N., West S., Proudfoot N.J. (2006). Pause sites promote transcriptional termination of mammalian RNA polymerase II. Mol. Cell Biol..

[bib28] Gu H., Do D.V., Liu X., Xu L., su Y., Nah J.M., Wong Y., Li Y., Sheng N., Tilaye G.A. (2018). The STAT3 target Mettl8 regulates mouse ESC differentiation via inhibiting the JNK pathway. Stem Cell Rep..

[bib29] Hamperl S., Bocek M.J., Saldivar J.C., Swigut T., Cimprich K.A. (2017). Transcription-replication conflict orientation modulates R-loop levels and activates distinct DNA damage responses. Cell.

[bib30] Hamperl S., Cimprich K.A. (2014). The contribution of co-transcriptional RNA:DNA hybrid structures to DNA damage and genome instability. DNA Repair (Amst)..

[bib31] Hatchi E., Skourti-Stathaki K., Ventz S., Pinello L., Yen A., Kamieniarz-Gdula K., Dimitrov S., Pathania S., Mckinney K.M., Eaton M.L. (2015). BRCA1 recruitment to transcriptional pause sites is required for R-loop-driven DNA damage repair. Mol. Cell.

[bib32] Jakkaraju S., Zhe X., Pan D., Choudhury R., Schuger L. (2005). TIPs are tension-responsive proteins involved in myogenic versus adipogenic differentiation. Dev. Cell.

[bib33] Jenjaroenpun P., Wongsurawat T., Sutheeworapong S., Kuznetsov V.A. (2017). R-loopDB: a database for R-loop forming sequences (RLFS) and R-loops. Nucleic Acids Res..

[bib34] Jenjaroenpun P., Wongsurawat T., Yenamandra S.P., Kuznetsov V.A. (2015). QmRLFS-finder: a model, web server and stand-alone tool for prediction and analysis of R-loop forming sequences. Nucleic Acids Res..

[bib35] Jentsch S., Psakhye I. (2013). Control of nuclear activities by substrate-selective and protein-group SUMOylation. Annu. Rev. Genet..

[bib36] Kogoma T., Hong X., Cadwell G.W., Barnard K.G., Asai T. (1993). Requirement of homologous recombination functions for viability of the Escherichia coli cell that lacks RNase HI and exonuclease V activities. Biochimie.

[bib37] Li M., Pokharel S., Wang J.T., Xu X., Liu Y. (2015). RECQ5-dependent SUMOylation of DNA topoisomerase I prevents transcription-associated genome instability. Nat. Commun..

[bib38] Li X., Manley J.L. (2006). Cotranscriptional processes and their influence on genome stability. Genes Dev..

[bib39] Manzo S.G., Hartono S.R., Sanz L.A., Marinello J., de Biasi S., Cossarizza A., Capranico G., Chedin F. (2018). DNA Topoisomerase I differentially modulates R-loops across the human genome. Genome Biol..

[bib40] Nadel J., Athanasiadou R., Lemetre C., Wijetunga N.A., Ó Broin P., Sato H., Zhang Z., Jeddeloh J., Montagna C., Golden A. (2015). RNA:DNA hybrids in the human genome have distinctive nucleotide characteristics, chromatin composition, and transcriptional relationships. Epigenetics Chromatin.

[bib41] Napoli S. (2013). Detailed analysis of promoter-associated RNA. Methods Mol. Biol..

[bib42] Nguyen H.D., Yadav T., Giri S., Saez B., Graubert T.A., Zou L. (2017). Functions of replication protein A as a sensor of R loops and a regulator of RNaseH1. Mol. Cell.

[bib43] Parkinson G.N., Lee M.P., Neidle S. (2002). Crystal structure of parallel quadruplexes from human telomeric DNA. Nature.

[bib44] Phan A.T., Gueron M., Leroy J.L. (2000). The solution structure and internal motions of a fragment of the cytidine-rich strand of the human telomere. J. Mol. Biol..

[bib45] Psakhye I., Jentsch S. (2012). Protein group modification and synergy in the SUMO pathway as exemplified in DNA repair. Cell.

[bib46] Roundtree I.A., Evans M.E., Pan T., He C. (2017). Dynamic RNA modifications in gene expression regulation. Cell.

[bib47] Salas-Armenteros I., Perez-Calero C., Bayona-Feliu A., Tumini E., Luna R., Aguilera A. (2017). Human THO-Sin3A interaction reveals new mechanisms to prevent R-loops that cause genome instability. EMBO J..

[bib48] Salvi J.S., Mekhail K. (2015). R-loops highlight the nucleus in ALS. Nucleus.

[bib49] Sanz L.A., Hartono S.R., Lim Y.W., Steyaert S., Rajpurkar A., Ginno P.A., Xu X., Chedin F. (2016). Prevalent, dynamic, and conserved R-loop structures associate with specific epigenomic signatures in mammals. Mol. Cell.

[bib50] Schlenk F., Depalma R.E. (1957). The formation of S-adenosylmethionine in yeast. J. Biol. Chem..

[bib51] Skourti-Stathaki K., Kamieniarz-Gdula K., Proudfoot N.J. (2014). R-loops induce repressive chromatin marks over mammalian gene terminators. Nature.

[bib52] Skourti-Stathaki K., Proudfoot N.J. (2014). A double-edged sword: R loops as threats to genome integrity and powerful regulators of gene expression. Genes Dev..

[bib53] Skourti-Stathaki K., Proudfoot N.J., Gromak N. (2011). Human senataxin resolves RNA/DNA hybrids formed at transcriptional pause sites to promote Xrn2-dependent termination. Mol. Cell.

[bib54] Smith J.J., Deane N.G., Wu F., Merchant N.B., Zhang B., Jiang A., Lu P., Johnson J.C., Schmidt C., Bailey C.E. (2010). Experimentally derived metastasis gene expression profile predicts recurrence and death in patients with colon cancer. Gastroenterology.

[bib55] Stork C.T., Bocek M., Crossley M.P., Sollier J., Sanz L.A., Chedin F., Swigut T., Cimprich K.A. (2016). Co-transcriptional R-loops are the main cause of estrogen-induced DNA damage. Elife.

[bib56] Takahara T., Tasic B., Maniatis T., Akanuma H., Yanagisawa S. (2005). Delay in synthesis of the 3' splice site promotes trans-splicing of the preceding 5' splice site. Mol. Cell.

[bib57] Tuduri S., Crabbe L., Conti C., Tourriere H., Holtgreve-Grez H., Jauch A., Pantesco V., de Vos J., Thomas A., Theillet C. (2009). Topoisomerase I suppresses genomic instability by preventing interference between replication and transcription. Nat. Cell Biol..

[bib58] Xu L., Liu X., Sheng N., Oo K.S., Liang J., Chionh Y.H., Xu J., Ye F., Gao Y.G., Dedon P.C., Fu X.Y. (2017). Three distinct 3-methylcytidine (m(3)C) methyltransferases modify tRNA and mRNA in mice and humans. J. Biol. Chem..

[bib59] Yang X., Liu Q.L., Xu W., Zhang Y.C., Yang Y., Ju L.F., Chen J., Chen Y.S., Li K., Ren J. (2019). m(6)A promotes R-loop formation to facilitate transcription termination. Cell Res..

[bib60] Yeon S.Y., Jo Y.S., Choi E.J., Kim M.S., Yoo N.J., Lee S.H. (2018). Frameshift mutations in repeat sequences of ANK3, HACD4, TCP10L, TP53BP1, MFN1, LCMT2, RNMT, TRMT6, METTL8 and METTL16 genes in colon cancers. Pathol. Oncol. Res..

[bib61] Yu K., Chedin F., Hsieh C.L., Wilson T.E., Lieber M.R. (2003). R-loops at immunoglobulin class switch regions in the chromosomes of stimulated B cells. Nat. Immunol..

[bib62] Zhang Z.Z., Pannunzio N.R., Hsieh C.L., Yu K., Lieber M.R. (2015). Complexities due to single-stranded RNA during antibody detection of genomic RNA:DNA hybrids. BMC Res. Notes.

[bib63] Zheng L., Roeder R.G., Luo Y. (2003). S phase activation of the histone H2B promoter by OCA-S, a coactivator complex that contains GAPDH as a key component. Cell.

[bib64] Zhou C., Molinie B., Daneshvar K., Pondick J.V., Wang J., van Wittenberghe N., Xing Y., Giallourakis C.C., Mullen A.C. (2017). Genome-wide maps of m6A circRNAs identify widespread and cell-type-specific methylation patterns that are distinct from mRNAs. Cell Rep..

