# Supplementary material for: The SUMOylated METTL8 Induces R-loop and Tumorigenesis via m3C
Source: iScience. 2020 Mar 7;23(3):100968. doi: 10.1016/j.isci.2020.100968 (PMC7082549; doi:10.1016/j.isci.2020.100968)
Supplement: Document S1. Transparent Methods and Figures S1–S3 [file mmc1.pdf]

iScience, Volume *23*

Supplemental Information

The SUMOylated METTL8 Induces

R-loop and Tumorigenesis via m3C

Li-Hong Zhang, Xue-Yun Zhang, Tao Hu, Xin-Yun Chen, Jing-Jia Li, Manfred Raida, Ning Sun, Yan Luo, and Xiang Gao

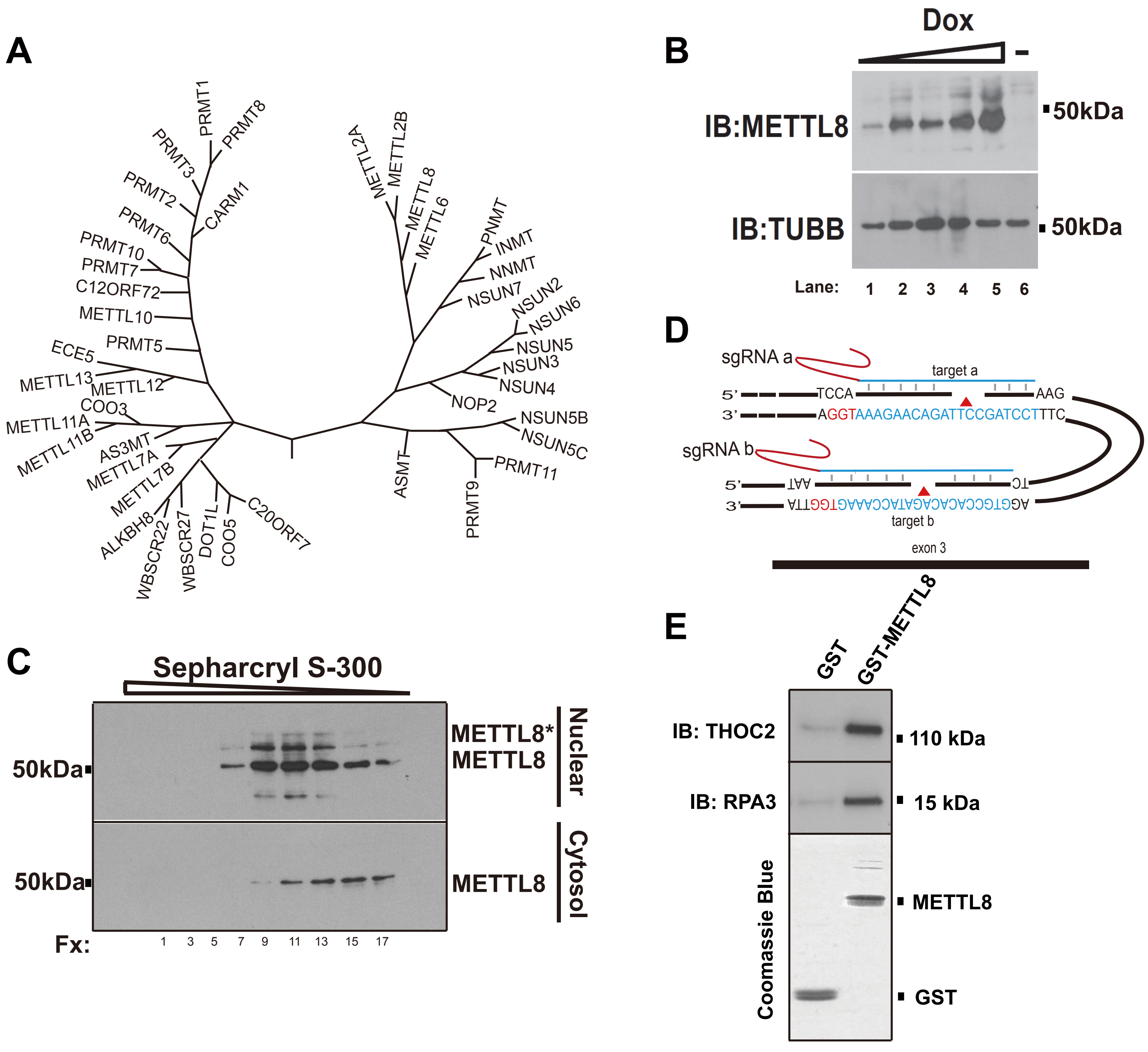

Figure S1. METTL8 protein family and its co-purification of binding partners, Related to Figure 1 (A) The distance between each methyltransferase was computed based on their primary protein sequence and plots were generated using MEGA 7.0.14 software. (B) The concentration of Doxycycline was titrated at 0.125 μg/ml so that ectopic induced METTL8 is close to endogenous protein level (lane 1). (C) Gel filtration (Sepharcryl S-300) separation of nuclear and cytosol METTL8 complex, alternative fractions were loaded onto gel and visualized with anti-METTL8 monoclonal antibody (Sigma, Atlas antibodies). (D) Schematic representation of the genomic locus of the double-nickase CRISPR/Cas9-mediated METTL8 knock out (top, with 2 sgRNAs targeting opposite DNA strands on the 3rd exon). (E) GST-pulldown assay with purified GST-METTL8 fusion protein and the GST protein; direct interaction with THOC2 and RPA3 was visualized with their respective antibody, protein molecular weight was indicated on right.

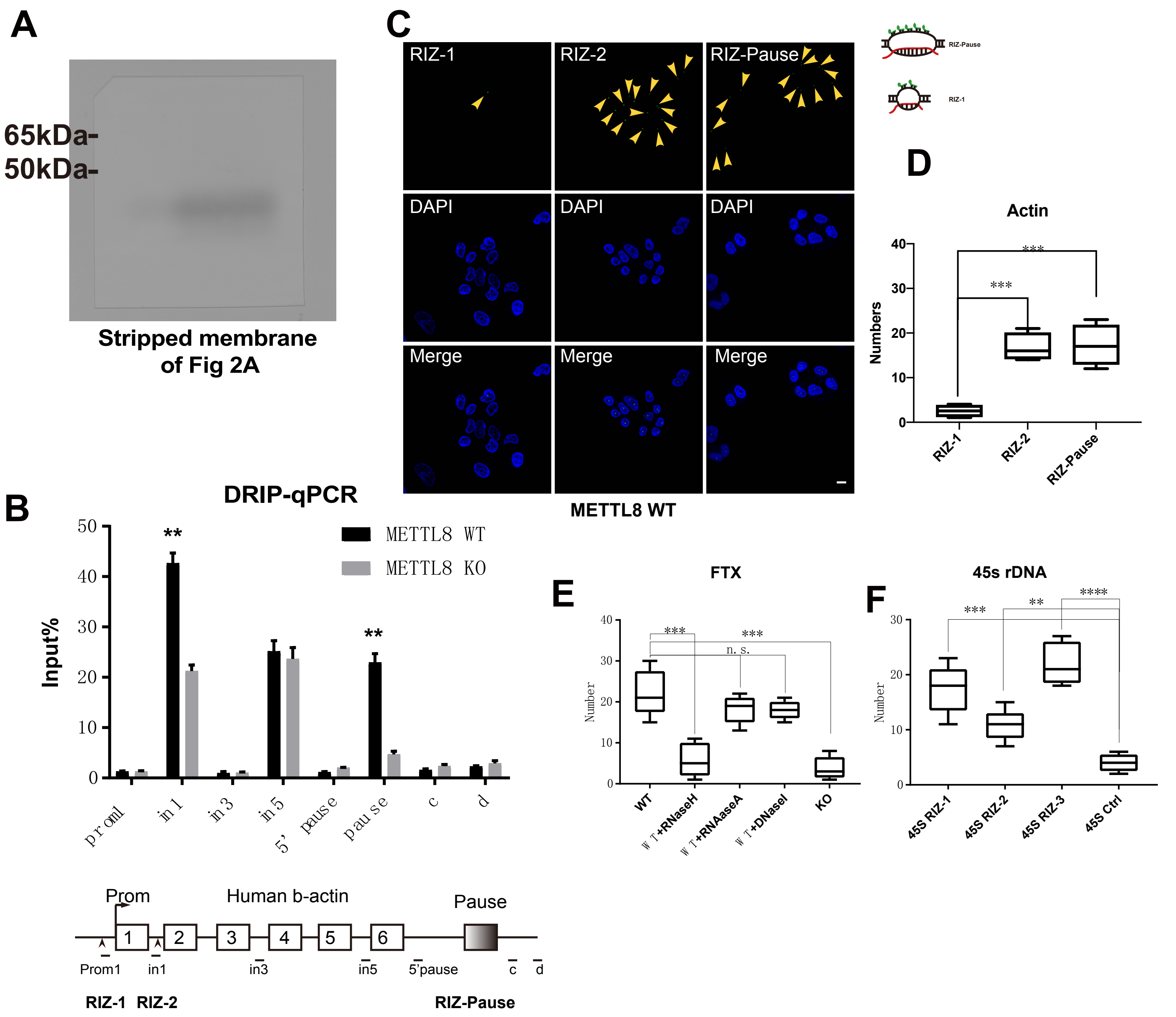

Figure S2. METTL8 regulates R-loop formation on the beta-actin gene, Related to Figure 2 (A) The stripped membrane of Figure 2A. (B) DRIP-qPCR analysis of HeLa METTL8 WT (black bars) and KO (gray bars); the number of R-loop binding sites (on beta-actin) was calibrated against the input nucleic acids; data are means ± SD (n=3, **P<0.01, Student's t-test). Bottom boxes, a schematic of human beta-actin gene dash lines indicating Prom 1, in 1, in3, in5, 5'pause and the pause sites. (C) FISH confocal microscopy imaging using the RIZ probes (green) indicated in (B, schematic on bottom); nuclei were counterstained using DAPI (blue). Scale bar, 10 μm. Arrowheads indicate R-loops detected by the FISH probes. (D) Statistic analysis of (C), stained dots (RIZ-1/2/Pause) were counted and data are mean ± SD, n=4, ****P<0.0001, Student's t-test. (E-F) Statistic analysis of Figure 2E and Figure 2G, respectively. Stained dots (R-loops) were counted and data are mean ± SD, n=4, **P<0.01, ***P<0.005, ****P<0.0001, Student's t-test.

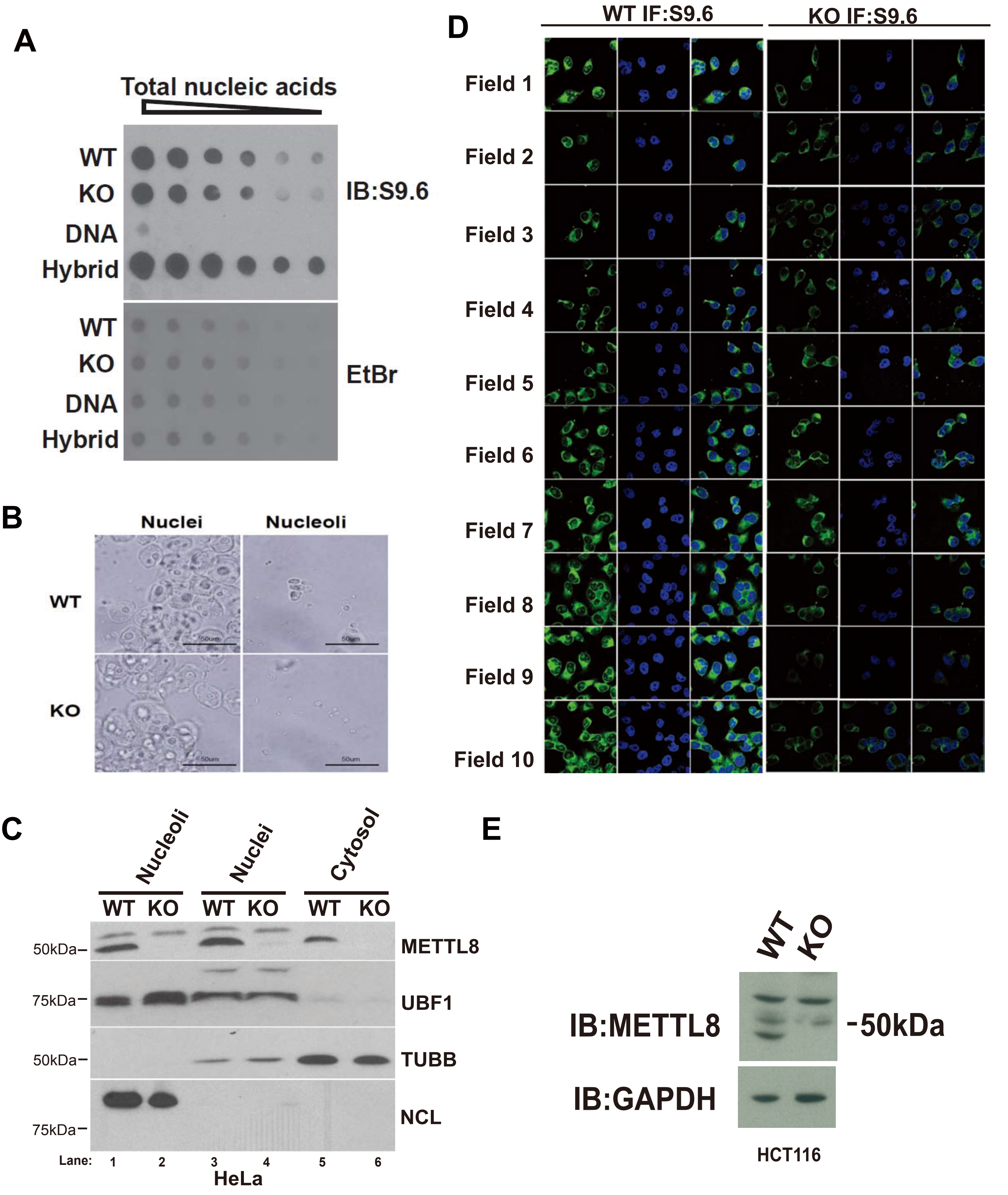

Figure S3. METTL8 regulates R-loops in the nucleolus, Related to Figure 3 (A) Dot blots display of R-loop structures from total HeLa cell nucleic acids. DNA (S9.6 negative, row 3) and DNA : RNA hybrid (S9.6 positive, row 4) were included for control. (B) Phase contrast microscopy showing purified HeLa nulei and nucleolus. Scale bar, 50 μm. (C) Western blots showing the fractioned HeLa METTL8 proteins from WT and KO cells, including cytosol (TUBB), nucleus (UBF1) and nucleolus (NCL) markers as controls, the band for METTL8 is below the 50 kDa marker. (D) Confocal imaging of R-loops (green) with S9.6 antibody, DAPI were included to counter-stain HeLa nucleus. 10 different view fields were taken and statistical analysis was performed on R-loops positive and negative cells (Figure 3F). Scale bar, 10 μm. (E) Western blots of HCT116 METTL8 proteins from WT and KO cells, GAPDH was included for loading control.

Transparent Methods

CRISPR/Cas9 directed knock out

Double-nikase CRISPR-Cas9 mediated knockout cells was done following the instruction of published protocols (Ran et al., 2013). Briefly, guiding RNA targeting to human METTL8 coding region was designed based on instructions on Feng Zhang's Target Finder (http://crispr.mit.edu). The two single guide RNAs with sequence: AAAGAACAGATTCCGATCCT, GTGCCACACAGATACCAAAG were cloned into PX462 vector based on the probability of the off-targets and their predicted on-site activity. Cas9 GFP expression was monitored 24h post transfection and positive clones were handpicked after 20 days of selection under puromycin (8 µg/mL). Cells were then expanded in the absence of puromycin to avoid the generation of any heterogeneity due to additional cutting, and clones screened by western blotting and immunofluorescence. Off-target site analysis of sgRNAs to identify potential off-target sites in the human genome, each sgRNA was BLASTed against the human genome (hg19). All sgRNAs of specified length to the sgRNA 3' end that conform to the NGG PAM used in experiments contains zero possible off-target.

Dot blot analysis

Dot blot was performed according to BioRad Bio-Dot Module (Product No.: 1706547 BioRad) instruction. Briefly, purified nucleic acids (DNA, RNA, R-loops) were treated accordingly (keep on ice or at 37-50℃ water bath) was loaded onto a nylon membrane (Product No.: 11209272001 Roche) using BioRad Bio-Dot Module connected to a vacuum, and then the DNA was crossed-linked using UV crosslinker (Stratalinker 2400, Stratagene). The DNA/RNA hybrid content was visualized using anti-DNA/RNA hybrid antibody (S9.6), while loading control was visualized under UV after ethidium bromide (10 µg/ml) staining.

Dot densitometry and Line scan analysis

Image pixel quantification was performed using ImageJ particle analysis function (area) and Line Profile Tool plugin (intensity), each dot/line (respectively) were scanned at least 3 times and image area/intensity values were averaged and standard deviations were calculated, graphs were drawn using Microsoft Excel.

Fluorescence *In situ* Hybridization (FISH)

FITC tagged DNA probes are synthesized commercially (Generay Biotechnology). R-loops Initiation Zoom (RIZ) sequences (complete list in Table S5) were predicted by the QmRLFS algorithm in R-loop DB (http://rloop.bii.a-star.edu.sg/). FITC tagged RIZ probes were reconstituted in hybridization buffer (10% Dextran Sulfate, 50% Formamide, 2×SSC, 1 mM EDTA, 50 mM Sodium Phosphate buffer pH 7.0). Cells were fixed in 4% PFA (25℃, 10 min) and permeabilized at least one hour or overnight in 70% ethanol (4℃) before incubating at 37℃ (overnight, in a humidified chamber) with 5 µl 50 mM pre-denatured (85℃, 10 min) RIZ probes. Then the cells were washed 3 times with 50% Formamide, 2×SSC for 5 min each at room temperature to remove all unhybridized or partially hybridized probes. Mount the coverslip on a slide with mounting media containing DAPI (Product No.: H-1200, Vector Laboratories) and fix it in place with a minimal amount of nail varnish and proceed to fluorescence imaging immediately.

Laser confocal microscopy

All confocal imagining were performed on Nikon A1R confocal microscope with ×60 object oil lens. A combination laser wavelength of 405/488/514 nm was used to detect DAPI/FITC/YFP

respectively. The image was analyzed on NIS-Elements Viewer 4.20 and exported to Adobe Illustrator for figure arrangement.

Cell nucleolar isolation

Nucleoli were prepared from HeLa S3, HCT116 cells following the LamondLab.com Nucleolar Isolation Protocol with some modification. Briefly, 10^7 cells were harvest by trypsinization followed by 3× wash with cold PBS. Then cells were resuspended in 3 mL Hypotonic Buffer A (10 mM Hepes, pH 7.9, 10 mM KCl, 1.5 mM $MgCl_2$, 0.5 mM DTT) and swelled on ice for 5 minutes. Swelled cells were checked under a phase contrast microscope and subjected to homogenization (10 strokes) using glass Douncer (Pestle "B", Wheaton Scientific). Homogenized cells were centrifuged at 2000 g for 10 min at 4℃, and supernatants (cytoplasm) and pellets (nuclei) were saved for analysis. 90% of the pellets containing nuclei were subject to nucleolar purification. Nuclei were resuspended in 1 mL S1 (0.25 M sucrose, 10 mM $MgCl_2$) and sonicated on ice for 6 × 5 seconds bursts with 5 seconds intervals between each burst (power setting 5, Misonix XL 2020). Sonicated nuclei were layered over 2 mL of S3 (0.88 M sucrose, 0.5 mM $MgCl_2$) and centrifuged at 3000 g for 10 min. The pellet contains nucleoli while the supernatant is retained as nucleoplasm.

Cell culture and generation of inducible protein complex system

HeLa S3 (ATCC CCL-2.2) cell line is maintained at 37℃, 5% CO_2 until 80% confluence before each passage. HeLa S3 suspension culture is achieved by inoculating exponential growth HeLa S3 cell into 10 liters of JMEM (Product No.: M0518 SIGMA) containing 10% FBS and 1% Pen-strep in a Corning (Product No.: CLS4500125 SIGMA) spinner flask. Inducible EF1a-FLAG-METTL8-HA stable cell line is generated by transfecting Clonetech (Cat. No.: 631337) TET-ON 3G vectors containing the EF1a-FLAG-METTL8-HA cassette with Blasticidine selection marker into HeLa S3 cell line, then the transfected HeLa S3 cells were maintained under 100 µM Blasticidine (Cat No.: A1113903 ThermoFisher Scientific) selection medium for two weeks or until colonies appeared. Positive colonies were handpicked and subjected to inducible expression profiling. Final Doxycyclin (Product No.: D3447 SIGMA) concentration for the endogenious level of METTL8 protein expression is deciphered at 0.125 µg/mL.

Liquid chromatography and purification scheme

1. DEAE anion column

DEAE Sepharose resin (Product No.: 17-0709-01) is purchased from GE Healthcare and poured into GE Healthcare xk 16/20 column, the resin bed is packed with a packing reservoir at a flow rate of 0.5 mL/min. After packing, the column is equilibrated with 2 times column volume of binding buffer. 10 mL of 10 mg/mL nuclear extract was loaded onto the DEAE column in low ionic strength buffer (10 mM Tris-HCl, 50 mM NaCl, pH 8.0) and the bounded proteins were step-wise eluted with buffer containing 100 mM, 300 mM, 500 mM and 1 M of NaCl. Each step-fraction was pooled and subjected to western blot analysis using antibody specified in the result section. The peak contains the highest amount of target protein was dialyzed with low ionic buffer overnight at 4℃ and subjected to the next step of purification.

2. Antibody NHS column generation

NHS-agarose-antibody column (Product No.:17-0716-01 GE Healthcare) is generated according to manufacturer's recommendation, anti-FLAG (Product No.: F1804, SIGMA) and anti-HA (Product No.: sc-7392, Santa Cruz) antibodies were purchased commercially.

3. NHS affinity column

Dialyzed DEAE fractions from previous steps were centrifuged to remove traces of precipitation before loading onto NHS-agarose-antibody column using a peristaltic pump. To ensure maximum antibody-antigen binding capacity, the sample was repeatedly passed through the antibody column at a flow rate of 0.2 mL/min for up to 16 hrs at 4℃. After applying the sample with the peristaltic pump, the charged column is linked to an ÄkTA FPLC system for wash and elution steps of the immune-captured protein complex. The wash steps include 2 CV of binding buffer (10 mM Tris-HCl, 50 mM NaCl, pH 8.0) equilibration and 2 CV of low ionic strength buffer (10 mM Tris-HCl, 100 mM NaCl, pH 8.0), after the wash, the complex is eluted with buffer containing 500 mM NaCl.

4. Gel filtration column

Gel filtration (Superose 6 10/300 GL GE Healthcare) column was used to characterize the native size of the immune-purified protein complex, and Sephacryl S-300 16/60 (GE Healthcare) was used to prepare protein fractions from the nuclear and cytosolic compartments. Sample was loaded onto the column and eluted with 1.5 CV isotonic buffers (10 mM Tris-HCl, 150 mM NaCl, pH 8.0).

5. Mono S strong cation column

Mono S strong cation exchange column was chosen for the final polishing step. The column is first equilibrated with binding buffer (10 mM Phosphate, 10 mM NaCl, pH 6.0), and sample dialyzed in the same buffer was loaded on to the column with ÄkTA FPLC system. The complex was eluted using a stepwise method. A salt gradient ladder of 30 mM, 100 mM, 300 mM, and 500 mM was pumped into the column and the complex is collected at 500 mM.

<u>Western blotting and silver staining</u>

All western blots were done according to BioRad Mini-PROTEAN and Mini Trans-Blot electrophoresis Transfer Cell protocol. Silver staining was also done with BioRad Silver Stain Plus kit.

<u>Mass-spectrometry</u>

A FASP (filter-aided sample preparation) aided mass-spectrometry was incorporated downstream of the complex purification. Peptides from the protein complex were extracted and solubilized and loaded onto FASP columns, samples were then alkylated, washed and digested on FASP Protein Digestion kit (Product No.: 44250 EXPEDEON). Digested peptides were then eluted, and dissolved in 0.1% formic acid in water. The eluted peptides were next submitted to nano-flow HPLC coupled to a QTOF mass spectrometer (1260 nano HPLC Agilent Technologies).

Raw Mass-spectrometry data were directly loaded onto PEAKS software and database search was performed against human IPI database (version 3.65), Carboxymethylated Cys was set as fixed modification, oxidized Met, deamidation of Asn and Gln, pyroGlu formation of the N terminus and acetylation of the N terminus as variable modification.

<u>Expression and purification of recombinant proteins</u>

To obtain purified, active proteins for *in vitro* enzyme assays, 6*HIS-SUMO1, 6*HIS-METTL8, 6*HIS-METTL8-K80R and 6*HIS-SUMO1gg were expressed in BL21 E. coli. at 20℃ under 100 µM IPTG induction for a period of 10~16 hours. The cells were then harvested by centrifugation and resuspended in cold PBS and sonicated twice at 10 Watts 5 mins to dissolve the recombinant proteins. The proteins were pre-cleared by centrifugation and

passing through empty agarose column which helps to remove background contaminants. The pre-cleared protein lysates were loaded either onto Gstrap or Histrap (GE healthcare) columns and the bounded recombinant proteins were eluted with 10 mM glutathione and 0.2 M imidazole respectively. The purified proteins were dialyzed against 10 mM Tris-HCl (pH 7.9) + 3 mM $MgCl_2$ to remove Imidazole and Glutathione. Protein concentration is determined by UV absorbance and snaps frozen in liquid nitrogen for storage up to 2 months.

In vitro sumoylation and methylation assay

In vitro sumoylation is carried out using SUMOLink SUMO-1 Kit (Product No.: 40120 ACTIVE MOTIF). Briefly, a master mix of 2 µg GST-UBA2, 1 µg GST-AOS1 and 0.5 µg 6*HIS-UBC9, is mixed with 3 µg of 6*HIS-SUMO1/1aa (non-conjugatable form) and 1~10 µg of METTL8, METTL8 complex or its K80R mutant. These enzymes are then incubated in a buffer containing a final concentration of 10 mM ATP at 37℃ for 1 hour. The reaction is stopped by adding ½ volume of SDS-PAGE sample buffers and boiled for western blots or further analysis.

In vitro methylation is carried out with a master mix of 2 µl S-adenosyl-L-[methyl-3H]-methionine, 2 g of METTL8, METTL8 complex, METTL8-ΔSAM, METTL8-K80R or DNMT1 protein, in combination with either METTL8 WT and KO HeLa nucleoli, or with purified DNA and KO from HeLa cells. Components are incubated in Histone methyltransferase buffer (25 mM Tris-Cl at pH 8.0, 20% glycerol) at 37℃ for 1 hour, and the reaction is either stopped by adding ½ volume of 4× SDS-PAGE sample buffer and boiled for SDS-PAGE separation, or proceed to Dot Blot analysis and liquid scintillation counter (Tri-Carb 2810TR, PerkinElmer).

PAR-CLIP and RNA immunoprecipitation

Photoactivated-ribonucleoside-enhanced crosslinking and immunoprecipitation (PAR-CLIP) is used for identifying METTL8 binding RNAs, the detailed procedure is published earlier (Hafner et al., 2010). Briefly, we incorporated photoreactive ribonucleoside analogs into nascent RNA transcripts in living cells, and irradiated the cells with UV at 365 nm inducing efficient crosslinking of the photoreactive nucleoside-labeled RNAs to interact with RNA binding proteins (RBPs), then the FLAG-tagged METTL8 protein complex is immunoprecipitated from the photo-activated cells and the crosslinked and co-immunoprecipitated RNAs were isolated. The isolated RNAs were then reverse-transcribed into cDNA library and subjected to Illumina Hiseq 2000 deep sequencing, raw sequencing data were mapped to the human genome version hg19 by Bowtie 1.0.0, METTL8 binding RNAs were listed in Table S2. For experimental control, the same procedure was repeated in METTL8 KO cells with FLAG antibody expressing FLAG vector, background binding RNAs were mapped to the hg19 and listed in Table S3.

DRIP-qPCR

DNA:RNA (S9.6) immunoprecipitation were performed followed by qPCR. Briefly, 10 µg S9.6 anti-R-loops antibody (Kerafast 1 µg/µl ENH001) were incubated with 5 µg of fragmented DNA in a 1.5 ml tube containing 40 µl of prewashed protein A beads, mix overnight in 4℃. Beads were washed the next day with TE buffer supplemented with 0.05% Tween, purify DNA using Qiagen MinElute kit. qPCR were performed using 2x SYBR premix (Takara RR820L), 0.2 l of 10 µM primers (Primer list) were mixed with 2 µl of S9.6-immunoprecipitate or 2 µl of 1:10 input chromatin.

Dual Luciferase assay

Either Wild-type or Mutant (C to G in m3C RIZ motif) *ACTB* pause regions were cloned into dual luciferase reporter vector with pGL4 backbone (Promega). The DNA sequence for the Wild-type *ACTB* pause region is GGGGAGTTCCTGGCCTGGCCCTTCTATGTCTCCCCAGGTACCCCAGTTTTTCTGGGTTCACCCAGAGTGCAG ATGCTTGAGGAGGTGGGAAGGGACTATTTGGGGGTGTCTGGCTCAGGTGCCATGCCTCACTGGG, whereas the Mutant pause region is GGGGAGTTCCTGGCCTGGCCCTTCTATGTCTCCCCAGGTACCCCAGTTTTTCTGGGTTCA**GGG**AGAGTG**G**A GATG**G**TTGAGGAGGTGGGAAGGGA**G**TATTTGGGGGTGTCTGGCTCAGGTGCCATGCCTCACTGGG, C to G mutations are underlined in bold. The self-cleaving ribozyme gene is subcloned from p370HU (Addgene 27386), and the IRES is subcloned from pIRES2-EGFP (Clontech). These reporter minigenes are then transfected into WT and METTL8 KO HCT116 cells in 24-wells at 90% confluence. Luciferase measurement was performed with standard protocol (Promega), briefly, the transfected cells were lysed in Passive Lysis buffer 24 hours after transfection, 20 µl of cell lysate was transferred to silicon-coated tube containing 100 µl LAR II buffer, mixed by pipetting 2 or 3 times, and Firefly luciferase activity was recorded with a luminometer. Next, 100 µl of Stop & Glo Reagent was added and vortex briefly, Renilla luciferase activity was recorded with the luminometer. All measurements are repeated in triplicates, background luminescence were measured and subtracted from all samples with nontransfected control cells.

ImageJ analysis

Dotblots and microscope images were loaded onto ImageJ (64-bit Java 1.8.0) free software, images were adjusted to gray scale and threshold were adjusted to auto. For dotblots, the dot intensities were measured using “measure” under the “analyze” menu; for line intensity scan, a line were drawn on the image at the sites of interest and line intensity were measured using “line scan” plugin. All measurements were performed in triplicates.

Soft agar colony formation assay

SeaPlaque™ Agarose with low melting temperature (Lonza, 50100) was used in this assay. In triplicate manner, 1.5ml culture medium with 0.6% agarose was first plated into each well of a 6-well plate. After base agarose solidified, another 1.5ml of 0.4% agarose was plated on top in culture medium containing 2000 cells per well. After 20 days colonies grown in soft agar were stained by 1 mg/ml Thiazolyl Blue Tetrazolium Bromide (Sigma, M5655) dissolve in water and then scanned in Bio-Rad Gel Doc EZ system. A population with more than 50 cells was counted as one surviving colony using Quantity One software.

Xenograft assay

$1x10^5$ METTL8 WT and KO HCT116 cells overexpressing different mutants were mixed with 1/3 matrigel and injected subcutaneously into the left or right flank on the back of 6-week old female NOD-SCID mice. After 4 weeks, tumor tissue was dissected and photographed.

Supplemental References

Hafner, M., Landthaler, M., Burger, L., Khorshid, M., Hausser, J., Berninger, P.,

Rothballer, A., Ascano, M., Jr., Jungkamp, A. C., Munschauer, M., Ulrich, A., Wardle, G. S., Dewell, S., Zavolan, M. & Tuschl, T. 2010. Transcriptome -wide identification of RNA-binding protein and microRNA target sites by PAR-CLIP. Cell, 141, 129-41.

Ran, F. A., Hsu, P. D., Wright, J., Agawala, V., Scott, D. A. & Zhang, F. 2013. Genome engineering using the CRISPR-Cas9 system. Nat Protoc, 8, 2281-2308.
